# Target 95-95-95 during childhood and pre-adolescence in Latin America and the Caribbean

**DOI:** 10.15649/cuidarte.4186

**Published:** 2025-04-11

**Authors:** Camila Moraes Garollo Piran, Natan Nascimento de Oliveira, Mariana Martire Mori, Rosana Rosseto de Oliveira, Leslie Villarroel Yañez, Andrés Antonio Gutiérrez-Carmona, Marcela Demitto Furtado

**Affiliations:** 1 Universidade Estadual de Maringá, Maringá, Brazil. camilagarollo@gmail.com Universidade Estadual de Maringá Universidade Estadual de Maringá Maringá Brazil camilagarollo@gmail.com; 2 Universidade Estadual de Maringá, Maringá, Brazil. nat_oliveira98@hotmail.com Universidade Estadual de Maringá Universidade Estadual de Maringá Maringá Brazil nat_oliveira98@hotmail.com; 3 Universidade Estadual de Maringá, Maringá, Brazil. mari_mmori@hotmail.com Universidade Estadual de Maringá Universidade Estadual de Maringá Maringá Brazil mari_mmori@hotmail.com; 4 Universidade Estadual de Maringá, Maringá, Brazil. rosanarosseto@gmail.com Universidade Estadual de Maringá Universidade Estadual de Maringá Maringá Brazil rosanarosseto@gmail.com; 5 Universidade de Antofagasta, Antofagasta, Chile. leslie.villarroel@uantof.cl Universidade de Antofagasta, Antofagasta, Chile Universidade de Antofagasta Antofagasta Chile leslie.villarroel@uantof.cl; 6 Universidade de Antofagasta, Antofagasta, Chile. leslie.villarroel@uantof.cl Universidade de Antofagasta, Antofagasta, Chile Universidade de Antofagasta Antofagasta Chile leslie.villarroel@uantof.cl; 7 Universidade Estadual de Maringá, Maringá, Brazil. mdfurtado@uem.br Universidade Estadual de Maringá Universidade Estadual de Maringá Maringá Brazil mdfurtado@uem.br

**Keywords:** Child, Adolescent, HIV, Acquired Immunodeficiency Syndrome, Health Policy, Niño, Adolescente, VIH, Síndrome de Inmunodeficiencia Adquirida, Política de Salud, Criança, Adolescente, HIV, Síndrome de Imunodeficiência Adquirida, Política de Saúde

## Abstract

**Introduction:**

The Joint United Nations Programme on HIV/AIDS (UNAIDS) has set the 95-95-95 targets. It is expected that 95% of HIV-positive individuals will know their HIV status and of these, 95% will be on antiretroviral therapy (ART) and 95% will achieve viral suppression.

**Objective:**

To analyze the distribution and spatial autocorrelation of the 95-95-95 targets among children and adolescents living with HIV/AIDS in Latin America and the Caribbean.

**Materials and Methods:**

An epidemiological ecological study using data from AIDSinfo by the Joint United Nations Programme on HIV/AIDS (UNAIDS) on the 95-95-95 targets among children and adolescents between 2015 and 2022. The analysis was performed using Moran's spatial autocorrelation coefficient (global and local), considering a 5% significance level.

**Results:**

A total of 52,000 and 42,000 cases of HIV/AIDS among children and adolescents in Latin America and the Caribbean were analyzed, respectively, for the period from 2015 to 2022. Disparities in the targets were identified between countries, with significant spatial autocorrelation for the second target of 95, showing a value of 0.375 (p-value 0.017).

**Discussion:**

Among the 27 countries included in the study, only Guatemala met the first 95 target. To reach the goal, strategies are needed to increase access to HIV testing, with more accessible counseling and testing services.

**Conclusion:**

There are discrepancies between countries in Latin America and the Caribbean in meeting and increasing the percentage of the 95-95-95 targets. The study allowed the identification of priority areas for attention, highlighting the need for new strategies and policies tailored to each locality, assisting in the achievement of the targets.

## Resumen

**Introducción:**

El Programa Conjunto de las Naciones Unidas sobre el VIH/SIDA (ONUSIDA) ha establecido las metas 95-95-95. Se espera que el 95% de los individuos con VIH conozcan su estado serológico, de estos el 95% estén en TAR y de estos el 95% logren la supresión viral.

**Objetivo:**

Analizar la distribución y autocorrelación espacial de las metas 95-95-95 entre niños y adolescentes que viven con VIH/SIDA en América Latina y el Caribe.

**Materiales y métodos:**

Estudio epidemiológico, ecológico con datos de AIDSinfo del Programa Conjunto de las Naciones Unidas sobre el VIH/SIDA (ONUSIDA) sobre las metas 95-95-95 en niños y adolescentes entre 2015 y 2022. Para el análisis se utilizó el Coeficiente de Autocorrelación Espacial de Moran (Global y Local), considerando un nivel de significancia del 5%.

**Resultados:**

Se analizaron 52.000 y 42.000 casos de VIH/SIDA entre niños, niñas y adolescentes de América Latina y el Caribe, respectivamente, en el periodo 2015 y 2022. Se identificaron disparidades en las metas entre países y autocorrelación espacial significativa para la segunda meta 95 con un valor de 0,375 (p-valor 0,017).

**Discusión:**

De los 27 países incluidos en el estudio, sólo Guatemala cumplió la primera meta 95. Para lograr la meta se requieren estrategias que aumenten el acceso a las pruebas de VIH, con servicios de consejería y pruebas más accesibles.

**Conclusiones:**

Existe una discrepancia entre los países de América Latina y el Caribe en el cumplimiento y crecimiento del porcentaje de las metas 95-95-95. El estudio permitió identificar áreas prioritarias de atención, destacando la necesidad de nuevas estrategias y políticas para abordar la temática, dirigidas a cada localidad, contribuyendo al logro de las metas.

## Resumo

**Introdução:**

O Programa Conjunto das Nações Unidas sobre HIV/aids (UNAIDS) determinou as metas 95-95-95. Espera-se que 95% dos indivíduos com HIV conheçam seu status sorológico, destes 95% estejam em uso de TARV, e destes, 95% alcancem a supressão viral.

**Objetivo:**

Analisar a distribuição e autocorrelação espacial das metas 95-95-95 entre crianças e adolescentes vivendo com HIV/aids na América Latina e Caribe.

**Materiais e métodos:**

Estudo epidemiológico, do tipo ecológico com dados do AIDSinfo do The Joint United Nations Programme on HIV/AIDS (UNAIDS) sobre as metas 95-95-95 entre crianças e adolescentes entre 2015 e 2022. Para análise foi utilizado o Coeficiente de Autocorrelação Espacial de Moran (Global e Local), considerando o nível de significância de 5%.

**Resultados:**

Foram analisados 52.000 e 42.000 casos de HIV/aids entre crianças e adolescentes na América Latina e Caribe, respectivamente, no período de 2015 e 2022. Foram identificadas disparidades das metas entre os países e autocorrelação espacial significativa para o segundo alvo 95 com valor de 0,375 (p-valor 0,017).

**Discussão:**

Dos 27 países incluídos no estudo, apenas Guatemala cumpriu o primeiro alvo 95. Para alcançar a meta é necessário estratégias que aumentem o acesso aos testes de HIV, com serviços de aconselhamento e testagem mais acessíveis.

**Conclusões:**

Há discrepância entre os países da América Latina e Caribe no cumprimento e crescimento do percentual das metas 95-95-95. O estudo possibilitou a identificação de áreas prioritárias de atenção, evidenciando a necessidade de novas estratégias e políticas de enfrentamento direcionadas a cada local, auxiliando no cumprimento das metas.

## Introduction

The Sustainable Development Goals (SDGs) portray a multisectoral vision for human development until 2030. There is just under a decade left for progress towards the SDGs, and it is important to understand the risks to society, in general, resulting from the failure to achieve the ambitious goals and their targets, especially for the end of acquired immunodeficiency syndrome (AIDS)^1^. 

In this context, in the report entitled Fast Track: Ending the AIDS epidemic by 2030, published in 2014, the Joint United Nations Program on HIV/AIDS (UNAIDS) determined the 95-95-95 targets with the aim of reducing to zero new HIV infections, zero discrimination and zero AIDS-related deaths caused by the Human Immunodeficiency Virus (HIV)^2^,^3^,^4^ . 

In 2022, approximately 1.5 million children aged 0 to 14 were living with HIV/AIDS worldwide, with the largest proportion concentrated in sub-Saharan Africa, despite the great advances observed in the prevention of vertical transmission of HIV^5^. 

Thus, to achieve further progress, social determinants of health are essential to address these multiple health burdens, and all SDGs that support the health and well-being of children and teenagers require extensive work within and across sectors to ensure effective change for this population^6^,^7^. Thus, strategies for functional cure of pediatric HIV need to target children and teenagers with very early initiation of Antiretroviral Therapy (ART), long-term viral suppression, and immunocompetence^8^. 

In order to achieve objective 3 of target 3.3, which is to end the AIDS epidemic by 2030^9^, progress is essential towards the 95-95-95 targets, which aim for 95% of people living with HIV to know their diagnosis, 95% of whom are on ART and 95% of those on ART to achieve viral suppression. It is noteworthy that among children and teenagers these rates are well below the established target, due to their vulnerability to HIV infection and the context in which they live^2^. 

Monitoring progress towards achieving the strategic HIV/AIDS targets in Latin America and the Caribbean depends on quality data to identify and address gaps in service coverage, quality of care and adherence to ART. Analyzing diagnosis, ART use and viral suppression among children and teenagers can provide a better understanding of the current situation and inform programs up to 2030. Such information can also serve as evidence to improve public policies in countries and stakeholders that play a key role in achieving the global targets. 

Thus, the question is: Is it possible to meet the UNAIDS 95-95-95 targets among children and teenagers? To this end, the objective of this study is to analyze the spatial distribution and autocorrelation of the 95-95-95 targets among children and teenagers living with HIV/AIDS in Latin America and the Caribbean. 

## Materials and Methods

This is an epidemiological, ecological study with data from children and teenagers living with HIV in Latin America and the Caribbean between 2015 and 2022. 

**Figure 1:**
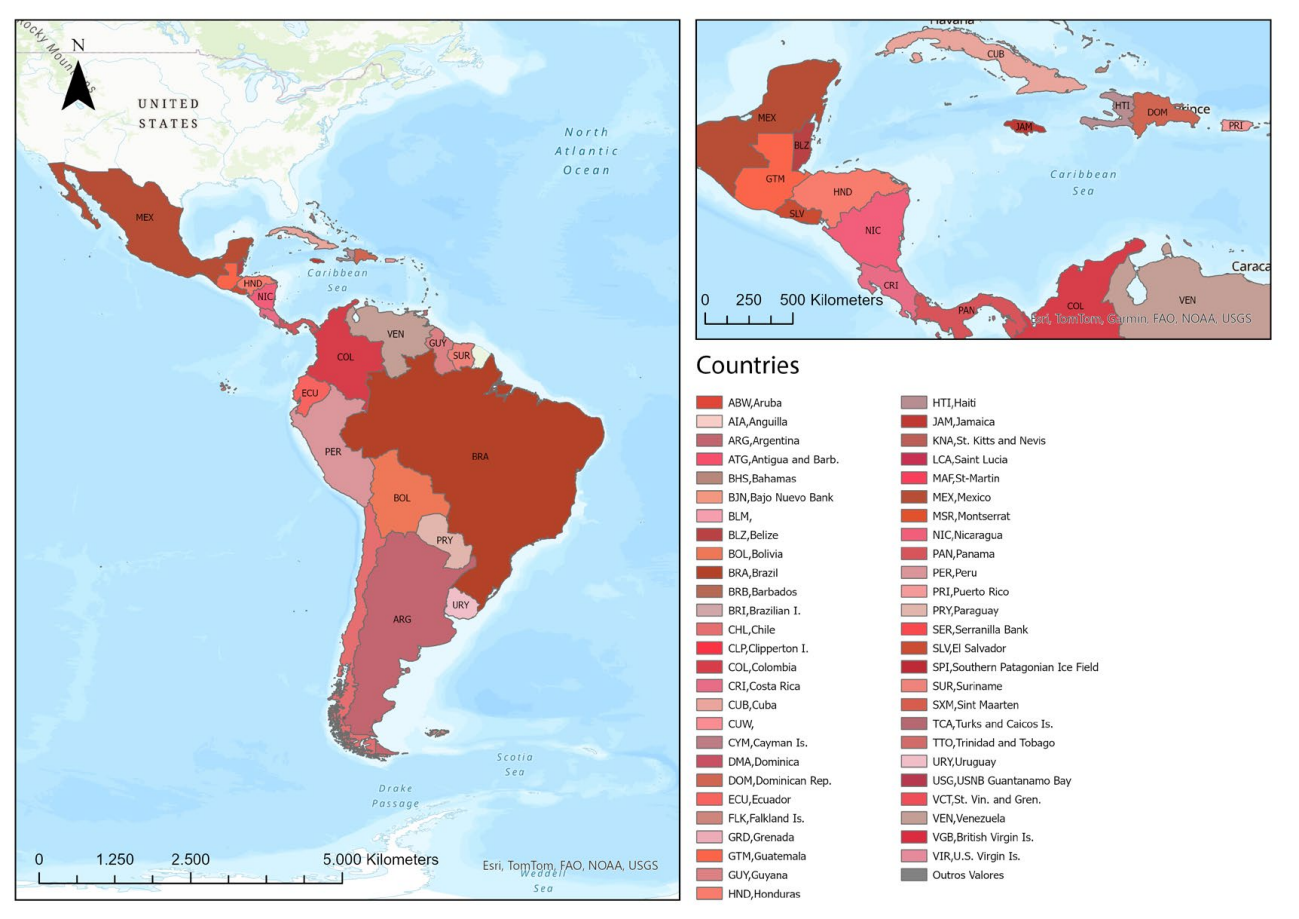
Distribution of Latin American and Caribbean countries. Antofagasta, Chile, 2024

*Source: Adapted from Economic Commission for Latin America and the Caribbean*

Latin America and the Caribbean (Figures 1) has an estimated population of 660 million inhabitants in 2022^10^. It is located between the Pacific Ocean and the Atlantic Ocean and has an extensive region that borders the United States of America. 

Records of HIV/AIDS cases among children and teenagers were extracted from The Joint United Nations AIDSinfo Program on HIV/AIDS (UNAIDS). All data are available at the UNAIDS website (https://aidsinfo.unaids.org/). The data was collected in December 2023. 

The variables used in the research were: country; compliance and growth of the 95-95-95 target among the age group of 0 to 14 years. According to the World Health Organization (WHO), childhood is considered as a period from 0 to 9 years, while teenage years is between 10 and 14 years of age^11^. 

Regarding countries with children and teenagers living with HIV that met and/or did not meet the 95-95-95 targets, we used the following definition: Percentage of people living with HIV who knew their HIV serological status (first target 95); Among people who knew their HIV serological status, percentage of those who had access to treatment (second target 95); And among people with access to treatment, percentage of those who achieved viral suppression (third target 95). 

The percentage change in targets between the first and last eight-year periods was calculated by subtracting the percentage of the last period from the percentage of the first period, dividing the result by the percentage of the first period, and multiplying the result by 100. The results regarding compliance with and growth of the 95-95-95 target among children and teenagers living with HIV/AIDS between 2015 and 2022 were presented in choropleth maps. Countries that met and showed growth in the percentage of the target were represented in green, while those that did not meet and decreased the percentage are in pink. 

Moran's Spatial Autocorrelation Coefficient was used for the statistical analysis of spatial dependence, which is subdivided into the Global Moran Index and the Local Moran Index. This statistic was used to analyze the spatial pattern of the variable according to the country. The value of the Global Moran Index varies between -1 and +1, so when the values are close to zero they indicate the absence of spatial autocorrelation, while values close to -1 or +1 indicate negative or positive autocorrelation^12^. 

To verify the significance, the pseudosignificance test with 999 permutations was adopted for the Global Moran Index. When the value was significant (p-value <0.05), the Local Moran Index, also called the Local Index Spatial Association, or LISA^12^, was applied. 

LISA allows the identification of clusters of areas with a similar risk of the expected outcome occurring, in other words, it allows verification to what extent the value of a variable is similar for a given area or different for its neighboring areas^12^. 

The clusters formed from the analysis of the Local Moran Index can be categorized as follows: high-high, when the countries and their neighbors had high rates; low-low, when the countries and their neighbors had low rates; low-high, for countries with low rates and neighbors with high rates; high-low, for countries with high rates and neighbors with low rates; and not significant (NS), when there was no clear spatial trend^12^. GeoDa software version 1.18 was used for statistical analyses, and QGIS software version 3.10 was used for choropleth maps. The data are stored in Mendeley Data^13^. 

As this is a study using data obtained from secondary sources, without naming the research subjects and whose access is in the public domain, there was no need for submission to the National Research Ethics Commission (Conep), in accordance with resolution no. 738, of February 1, 2024. 

## Results

**Figure 2:**
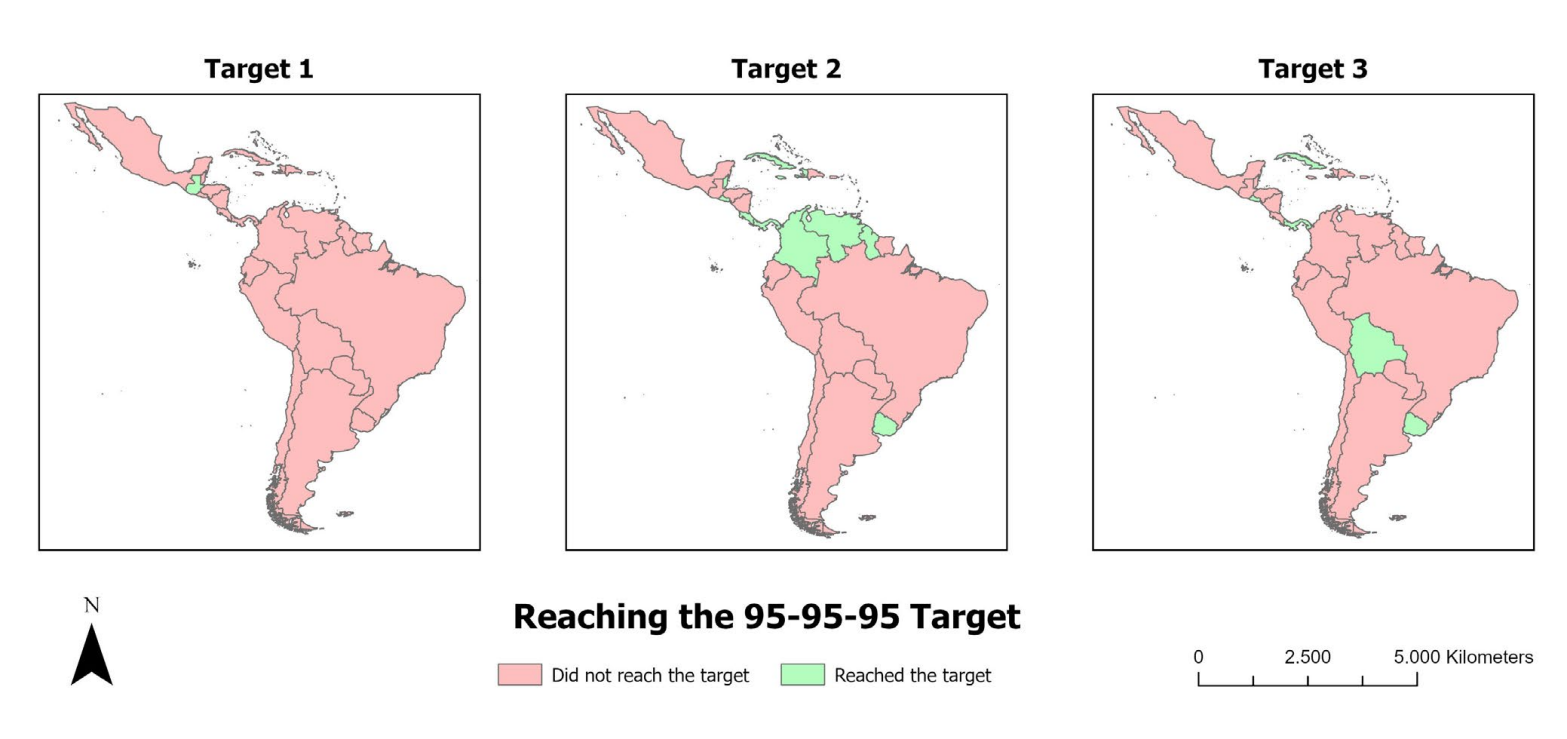
Spatial distribution of achievement of the 95-95-95 target among children and teenagers living with HIV/AIDS in Latin America and the Caribbean, between 2015 and 2022. Antofagasta, Chile, 2024

*Source: Prepared from data from AIDSinfo of The Joint United Nations Program on HIV/AIDS.*

A total of 52,000 and 42,000 cases of HIV/AIDS among children and teenagers in Latin America and the Caribbean, respectively, were analyzed, which occurred between 2015 and 2022. In the spatial distribution analysis regarding compliance with the 95-95-95 targets, disparities were found between countries. Regarding the first target of 95, only Guatemala achieved the target, while regarding the second target of 95, ten countries achieved the target. Regarding the third target of 95, only four countries achieved viral suppression among children and teenagers, namely Uruguay, Bolivia, Panama and Cuba (Figures 2). 

**Figure 3:**
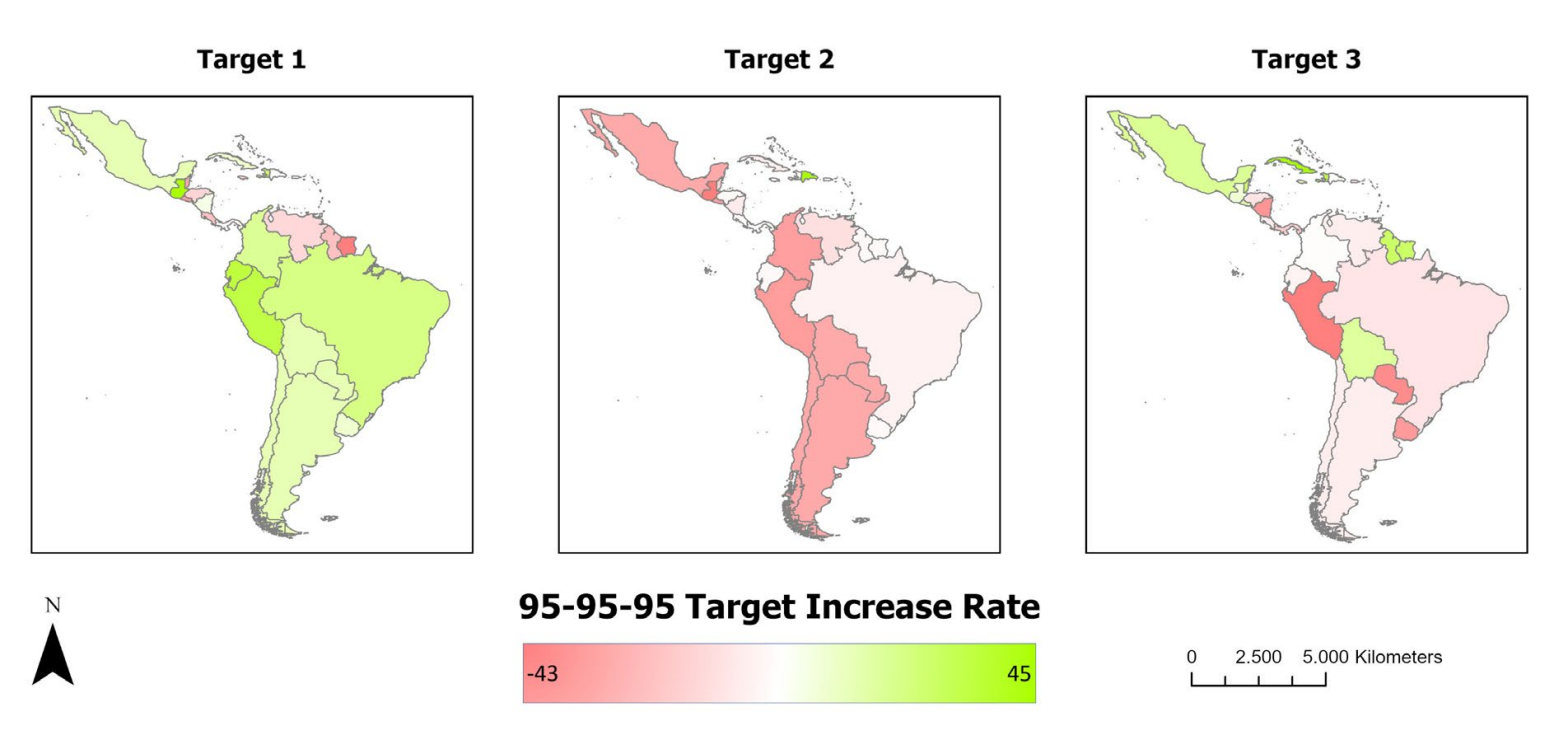
Spatial distribution of growth in the 95-95-95 target among children and teenagers living with HIV/AIDS in Latin America and the Caribbean, between 2015 and 2022. Antofagasta, Chile, 2024

*Source: Prepared from data from AIDSinfo of The Joint United Nations Program on HIV/AIDS.*

When observing the growth of the 95-95-95 targets, it was noted that 11 countries showed an increase in the first target 95, with the highest percentages in Guatemala (45.00%), Ecuador (34.00%) and Peru (33.00%) and the lowest percentage of growth was in Suriname (-43.00%). In the second target 95, only the Dominican Republic increased the percentage of achievement by 37%. In relation to the third target 95, eight countries showed an increase in the percentage, concentrated mainly in Cuba (34.00%), followed by Haiti (26.00%) (Figures 3). 

**Figure 4:**
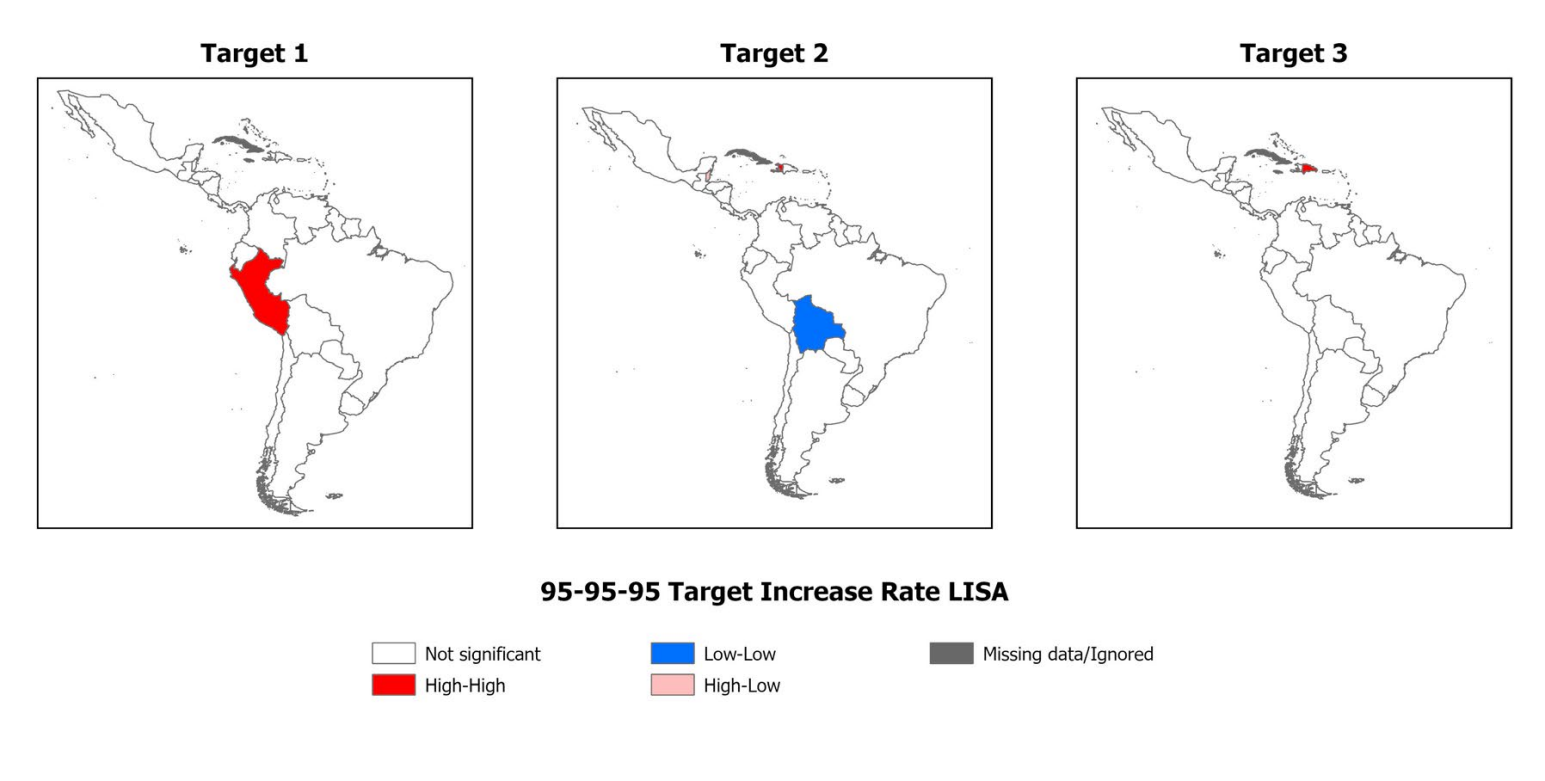
Spatial autocorrelation of the 95-95-95 target among children and teenagers living with HIV/AIDS in and around neighboring countries between 2015 and 2022. Antofagasta, Chile, 2024

*Source: Prepared from data from AIDSinfo of The Joint United Nations Program on HIV/AIDS.*

 Clusters were formed from clusters in countries that showed significance regarding the relationship between the 95-95-95 target and place of residence. The global Moran's Index showed significant spatial autocorrelation for the second target 95, with a value of 0.375 (p-value 0.017). In total, 24 countries did not demonstrate significance, with a Low-Low autocorrelation pattern being identified in Bolivia, High-Low in Belize and High-High in Haiti (Figures 4).

 For the first target 95 and third target 95, there was a Global Moran Index of 0.016 (p-value 0.760) and 0.190 (p-value 0.200), respectively, with a random pattern, not equivalent to the territory. In the Local Moran Index in the first target 95, Peru presented a High-High pattern and in the third target 95, only the Dominican Republic showed a High-High pattern (Figure 4).

## Discussion

This study stands out for its unprecedented spatial correlation of the 95-95-95 targets among children and teenagers with HIV/AIDS living in Latin American and Caribbean countries. Using spatial analysis tools, the study provides valuable information on the current state of the HIV epidemic, in addition to enabling an understanding of the relationship between the event and neighboring countries and the main geographic disparities; knowledge about positive serology; ART status and viral load suppression among HIV-positive children and teenagers. 

Of the 27 countries included in the study, only Guatemala met the first target 95. Given that many children and teenagers are infected with HIV through vertical transmission, the Guatemalan Ministry of Public Health and Social Welfare (MSPAS) stands out, recommending that, if HIV testing is available, pregnant women be tested at their first prenatal visit. And that pregnant women who are HIV-positive and on antiretroviral treatment should receive public health messages^14^. However, other countries also conduct testing during prenatal care but have not met the targets. 

Therefore, to achieve all the targets of the 95-95-95 goal, control HIV and end AIDS, it is necessary to act quickly to reduce the number of people who are unaware of their serological status, especially among children and adolescents, who are more susceptible to not meeting the targets. 

Therefore, it is urgent to develop tools and strategies to increase access to HIV testing, in addition to making HIV counseling and testing services more accessible and attractive to vulnerable groups^15^. 

It is believed that some administrative and legislative changes in many Latin American and Caribbean countries are necessary to meet the goals, as well as an increase in funds to expand testing strategies and programs to initiate ART on the same day of diagnosis to achieve viral suppression^15^.

There was an exponential and constant growth and decrease in compliance with the 95-95-95 targets, with the Andean corridor countries appearing at the epicenter of the decline in the second 95 target. This decrease can be justified, basically, due to the high migratory flow in which the implementation of HIV prevention, treatment and care programs is rarely considered^15^. 

Thus, the continuity of ART is threatened when the family of the child and/or teenager is migrating. Therefore, a coordinated plan is needed to ensure that people who migrate across borders can have their fundamental human right to health^15^. 

Countries receiving migrants from Venezuela and elsewhere, especially Argentina, Chile, Colombia, Mexico and Peru, must strengthen their health systems to meet the health care needs of refugees and migrants in order to minimize negative HIV-related consequences^15^. It is well known that cultural and political issues associated with structural vulnerability to HIV infection are embedded in global and local border processes^16^. 

Even with greater availability and access to ART in low and middle income countries, to achieve long-term virological suppression and meet the 95-95-95 targets, there is a major challenge in reducing the high prevalence of virological treatment failure among children and teenagers living with HIV/AIDS^17^. 

Therefore, it is necessary for countries to increase the use of HIV plasma viral load tests to monitor the response to ART and tests for resistance to antiretroviral drugs, which will consequently help in the immediate identification of the situation and patients in failure^17^. 

This study identified that Bolivia was a country that did not meet the second target 95 and was surrounded by neighboring countries that also did not reach this target and did not show growth in the target between 2015 and 2022. This fact may reflect the Corona Virus Disease 2019 (Covid-19) between the end of 2019 and 2022. Covid-19 affected adherence to ART, mainly in vulnerable groups such as children and teenagers, especially with regard to difficulty in accessing health services^18^. 

During this period, many HIV centers were converted into COVID-19 treatment centers, there were restrictions on public transportation, resource constraints for HIV treatment, as well as the reassignment of health professionals to contain the pandemic, and a reduction in the income of the general population due to the need for social isolation^18^. This context may have been a trigger for the non-compliance with the 95-95-95 targets both in Bolivia and in Latin America and the Caribbean as a whole. 

However, Haiti, in addition to meeting the second Target 95 goal, was also surrounded by neighbors who did. Haiti works with Multi-Month Dispensing (MMD) of ART is a promising strategy for improving adherence to treatment. Extending ART dispensing intervals increases the likelihood of retention for 12 months after starting treatment. Thus, in recent years, Haiti has achieved considerable success with HIV treatment through MMD, combined with health education activities to change sexual behavior and improvements in care delivery in rural and urban areas^19^. In addition, upon diagnosis, people receive counseling and start treatment. This strategy is being used to curb the pandemic of new HIV cases in the country and reduce the number of deaths^20^. 

Belize, despite being a country that lives with financial vulnerability, reached the second target of 95, even though it is surrounded by neighbors that have not progressed in relation to the goals, which indicates that the country is focused on eliminating the barriers that affect health care within its territory. 

A study conducted in 2016 in Belize showed that almost half of patients diagnosed with HIV/AIDS are lost to follow-up, and as one of the strategies to solve this problem, more health professionals were added to advise adherence to the system and reduce existing gaps. This position was created with the purpose of relieving the workload of social workers, who were previously solely responsible for ensuring adherence^21^1. 

Sex education is a tool that should be applied in different countries, especially with children and young people. It is a strategy capable of preventing new HIV infections, encouraging diagnosis, and consequently, raising awareness among infected people about the importance of undergoing the correct treatment, aiming for an undetectable viral load. In this sense, countries such as Uruguay and Cuba, which met the third target 95, have legislation that discusses sex education. In Uruguay, Law 18.987 of 2009 includes the approach of comprehensive sex education, focusing on gender and sexual rights. Meanwhile, in Cuba, there is resolution 139/2011, which deals with comprehensive sex education in Cuban schools, so that children and teenagers receive correct guidance^22^. 

Although in Latin America and the Caribbean, policies related to diagnosis and treatment have been adopted in more than 80% of these countries, it is noted that there are flaws that weaken HIV monitoring due to the lack of simultaneous integration of notifications, thus hindering the quality of care provided^23^. 

Despite all the strategies implemented by the countries studied, inequalities in treatment coverage mean that some population groups are left behind and vulnerable to continued HIV transmission. Furthermore, the effect of viral suppression on reducing cases will only be fully resolved when the goals of access to diagnosis and treatment are achieved among the population, including children and teenagers in different locations. In this sense, it is essential to focus on the individual, social and structural barriers that a given population faces, in order to guarantee access to services^24^. Even with progress in some countries, children and teenagers living with HIV are still disproportionately affected and thus fall behind in achieving these goals^2^,^3^,^4^. 

These results help in the development, strengthening and/or implementation of intersectoral policies between countries with the aim of transforming this scenario to achieve the 2030 goals, through the creation of unique strategies for the territories of Latin America and the Caribbean, as well as the construction of indicators sensitive to this context. 

One of the limitations of this research, in addition to those related to the use of secondary data subject to incomplete and/or erroneous filling, was the failure to identify research for comparability and greater in-depth discussion on the geographic distribution and spatial autocorrelation of the 95-95-95 targets in Latin America and the Caribbean. However, this study can support future epidemiological research on the spatial analysis of HIV/AIDS among children and teenagers and assist health managers in decision-making in these countries. 

## Conclusions

The study allowed us to analyze the spatial distribution and autocorrelation of the 95-95-95 targets among children and teenagers living with HIV/AIDS in Latin American and Caribbean countries. The spatial distribution showed disparities between countries in terms of meeting and increasing the achievement of the 95-95-95 targets. 

Furthermore, the clusters identified by LISA highlight priority areas of attention in Latin America and the Caribbean, pointing to the need for new strategies and policies that consider the specificities of each location to ensure the assertive targeting of actions and services that corroborate progress towards the 95-95-95 targets among children and teenagers in these countries. 

It was observed that disparities persist, especially in communities that face barriers to essential health services. Therefore, it is suggested that strategies focus on prevention, health education, and increased access to HIV testing and treatment to achieve short-term goals. In addition, they should address social determinants of health, reduce prejudice and stigma through awareness, and ensure equitable access to health care to achieve the ambitious goal of ending AIDS by 2030. 

For future developments, a multicenter study is suggested with managers from each Latin American and Caribbean country responsible for health policies focused on HIV, together with family members, children and teenagers, with the aim of better understanding the phenomenon, as this can reestablish a new plan of action. 
